# Development and validation of a risk score for hospitalization for heart failure in patients with Type 2 Diabetes Mellitus

**DOI:** 10.1186/1475-2840-7-9

**Published:** 2008-04-22

**Authors:** Xilin Yang, Ronald C Ma, Wing-Yee So, Alice P Kong, Gary T Ko, Chun-Shun Ho, Christopher W Lam, Clive S Cockram, Peter C Tong, Juliana C Chan

**Affiliations:** 1 Department of Medicine and Therapeutics, The Chinese University of Hong Kong, Hong Kong SAR, China; 2 Li Ka Shing Institute of Health Sciences, The Chinese University of Hong Kong, Hong Kong SAR, China; 3 Hong Kong Institute of Diabetes and Obesity, The Chinese University of Hong Kong, Hong Kong SAR, China; 4 Department of Chemical Pathology, The Chinese University of Hong Kong, Hong Kong SAR, China

## Abstract

**Background:**

There are no risk scores available for predicting heart failure in Type 2 diabetes mellitus (T2DM). Based on the Hong Kong Diabetes Registry, this study aimed to develop and validate a risk score for predicting heart failure that needs hospitalisation in T2DM.

**Methods:**

7067 Hong Kong Chinese diabetes patients without history of heart failure, and without history and clinical evidence of coronary heart disease at baseline were analyzed. The subjects have been followed up for a median period of 5.5 years. Data were randomly and evenly assigned to a training dataset and a test dataset. Sex-stratified Cox proportional hazard regression was used to obtain predictors of HF-related hospitalization in the training dataset. Calibration was assessed using Hosmer-Lemeshow test and discrimination was examined using the area under receiver's operating characteristic curve (aROC) in the test dataset.

**Results:**

During the follow-up, 274 patients developed heart failure event/s that needed hospitalisation. Age, body mass index (BMI), spot urinary albumin to creatinine ratio (ACR), HbA_1c_, blood haemoglobin (Hb) at baseline and coronary heart disease during follow-up were predictors of HF-related hospitalization in the training dataset. HF-related hospitalization risk score = 0.0709 × age (year) + 0.0627 × BMI (kg/m^2^) + 0.1363 × HbA_1c_(%) + 0.9915 × Log_10_(1+ACR) (mg/mmol) - 0.3606 × Blood Hb(g/dL) + 0.8161 × CHD during follow-up (1 if yes). The 5-year probability of heart failure = 1-S_0_(5)^EXP{0.9744 × (Risk Score - 2.3961)}^. Where S_0_(5) = 0.9888 if male and 0.9809 if female. The predicted and observed 5-year probabilities of HF-related hospitalization were similar (p > 0.20) and the adjusted aROC was 0.920 for 5 years of follow-up.

**Conclusion:**

The risk score had adequate performance. Further validations in other cohorts of patients with T2DM are needed before clinical use.

## Background

Besides coronary heart disease (CHD), diabetes is another major cause for hospital admissions due to heart failure (HF), which contributes to major morbidity and premature mortality in people with diabetes [1]. Subjects with Type 2 diabetes and impaired glucose regulation have 2.8-fold and 1.7-fold risk of developing HF respectively, when compared to individuals with normoglycemia [2]. The Framingham Study [3] and the United Kingdom Prospective Diabetes Study (UKPDS) developed risk scores or engines to predict CHD-related events [4] and stroke [5]. Based on the Hong Kong Diabetes Registry, our group has developed and validated risk scores for predicting end-stage renal disease [6,7], stroke [8], coronary heart disease [9] and all-cause mortality [10]. These risk equations may enable risk stratification for more effective preventive strategies in Chinese patients with type 2 diabetes. Notwithstanding the importance of HF in type 2 diabetes, the predictors for HF have not been fully explored.

The Hong Kong Diabetes Registry was established in 1995 as a quality assurance and continuous improvement tool with particular focus on risk stratification, treatment to targets and patient empowerment. In the present analysis, we aimed to develop and validate a risk score for predicting HF that needed hospitalization.

## Methods

### Subjects

The Prince of Wales Hospital is a regional hospital which covers a catchment area of 1.2 million residents. The Hong Kong Diabetes Registry was established in 1995 and enrols 30–50 ambulatory diabetic patients each week. The referral sources included general practitioners, community and other specialty clinics as well as patients discharged from hospitals. Enrolled patients with hospital admissions within 6–8 weeks prior to assessment accounted for less than 10% of all referrals. The 4-hour assessment of complications and risk factors was performed on an outpatient basis, modified from the European DIABCARE protocol [11]. The study was approved by the Chinese University of Hong Kong Clinical Research Ethics Committee and written informed consent was obtained from all patients. From 1995 to 2005, 7920 diabetic patients were enrolled in this Registry. Among them, 332 with Type 1 diabetes defined as acute presentation with diabetic ketoacidosis, heavy ketonuria (>3+) or continuous requirement of insulin within 1 year of diagnosis, and 5 with uncertain type 1 diabetes status, were excluded from the analysis. In addition, 49 with non-Chinese or unknown nationality were excluded. In line with the UKPDS CHD risk engine [4] and our CHD risk score [9], 467 patients were also excluded due to past history of CHD or HF. A total of 7067 Chinese patients with type 2 diabetes who were free of past history of HF and CHD at enrolment were included in this analysis.

### Clinical outcomes

Clinical endpoints included hospital discharge diagnoses and mortality recorded before or on 30^th ^July 2005 were recorded or otherwise censored on 30^th ^July 2005. Details of hospital admissions were retrieved from the Hong Kong Hospital Authority Central Computer System. The latter records admissions to all public hospitals, which account for about 95% of hospital bed-days due to the heavily subsidized health care system in Hong Kong. These databases including the Hong Kong Death Registry were matched by a unique identification number, the Hong Kong Identity Card number, which is compulsory for all residents in Hong Kong. Principal hospital discharge diagnoses coded by the International Classification of Diseases Ninth Revision were used to identify HF that needed hospitalisation (Code 428). The diagnosis of HF was consistent with the recommended guidelines [12]. Follow-up time was calculated as the time from enrolment to the first hospitalization for HF, death or 30^th ^July 2005.

### Clinical measurements

Details of assessment methods and definitions have been previously described [6,8-10,13]. On the day of assessment, patients attended the centre after 8 hours of fasting and underwent anthropometric measurements and laboratory investigations. Apart from documentation of demographic data and clinical assessment of complications, fasting blood samples were taken for measurement of plasma glucose, glycosylated haemoglobin (HbA_1c_), lipid profile (total cholesterol, high density lipoprotein cholesterol and triglycerides, calculated low density lipoprotein cholesterol, renal and liver functions. A sterile, random spot urine sample was used to measure albumin creatinine ratio (ACR). Peripheral arterial disease was defined by lower limb amputation, the absence of foot pulses on palpation, confirmed by an ankle:brachial ratio ≤0.90 as measured by Doppler ultrasound examination, or history of revascularization for peripheral arterial disease. This study used the abbreviated Modification of Diet in Renal Disease (MDRD) formula re-calibrated for Chinese [14] to estimate glomerular filtration rate (eGFR) expressed in ml min^-1 ^1.73 m^-2^: eGFR = 186 × [SCR × 0.011] ^-1.154 ^× [age] ^-0.203 ^× [0.742 if female] × 1.233, where SCR is serum creatinine expressed as μmol/l (original mg/dL converted to μmol/l) and 1.233 is the adjusting coefficient for Chinese ethnicity. Laboratory assays have also been described previously [6].

### Statistical analyses

The Statistical Analysis System (Release 9.10) was used to perform the statistical analysis (SAS Institute Inc., Cary, USA). Split-half validation was used to develop the risk score. The dataset was randomly divided into two datasets using a computer-generated random number: the training dataset for model development and the test dataset for validation of the developed HF risk score. In the training dataset, Cox proportional regression analysis with the backward algorithm (p < 0.05 for stay) was used to select predictors of HF. The candidate baseline variables for inclusion in the final model included age, sex, current and ex smoker status, duration of diabetes, systolic blood pressure, glycated haemoglobin (HbA_1c_), body mass index (BMI), blood haemoglobin (Hb), white blood cell count, high density lipoprotein cholesterol, low density lipoprotein cholesterol, triglyceride, non-high density lipoprotein cholesterol, spot urinary ACR, eGFR, sensory neuropathy, retinopathy, peripheral arterial disease, history of stroke, and drug use variables (lipid-lowering drugs and angiotensin-converting enzyme (ACE) inhibitors or angiotensin II receptor blockers (ARB), diet treatment, oral anti-diabetic drugs, other antihypertensive drugs, and insulin at enrolment). As CHD is an important risk predictor of HF [15], we added an additional variable that recorded CHD events during follow-up period [9] to improve the predicting ability of the HF risk score. Proportional hazards assumption and functional form were checked using Supremum test [16].

Construction of risk scores and probability equations from Cox models have been described previously [8,9]. The shrinkage factor was calculated using (LR-p)/LR, where LR denotes the likelihood ratio χ^2 ^and p the number of the predictors in the final model (below 0.85 raises concern of over-fitting) [17].

Validation of the risk score was performed using the test dataset. Calibration was checked using the Hosmer and Lemeshow test [10]. Overall discrimination was checked by C index as calculated by Pencina's method [18]. The 5-year discrimination as indicated by the area under receiver operating characteristic curve, as well as the 5-year sensitivity and specificity were calculated using Chambless' method [19]. A p value < 0.05 was considered to be statistically significant.

## Results

### Characteristics of study population

At enrolment, the median age of the cohort was 57 years (interquartile range, IQR: 46–67 years) and the median duration of diabetes was 5 (IQR: 1–11) years. During a median follow-up period of 5.52 years (IQR: 2.90–7.87 years), 3.32% (n = 274) of patients had HF that needed hospitalisation, giving an incident rate of 7.17 (95% CI: 6.33–8.02) per 1000 person-years. During the follow-up period, 681 patients died. Other population characteristics are listed in Table 1. In the training dataset, compared to patients without hospitalisation for HF, those with hospitalisation for HF had older age (median/IQR 69/13 vs. 56/20 years, p < 0.0001), higher HbA_1c _(7.8/2.6 vs. 6.7/2.1%, p < 0.0001), higher ACR (52.5/230.7 vs. 1.9/8.4 mg/mmol, p < 0.0001), lower blood Hb (12.5/2.6 vs. 13.9/2.1 g/dL, p < 0.0001) and longer duration of diabetes (10/11 vs. 5/9 years, p < 0.0001) but had similar BMI (24.7/4.9 vs. 24.7/4.8 kg/m^2^, p = 0.5021).

**Table 1:** Baseline clinical and biochemical characteristics of 7074 Chinese Type 2 diabetic patients without clinical evidence of coronary heart disease and heart failure at enrollment during 5.52 years of follow-up in the training dataset and the test dataset

	Training dataset (n = 3456)		Test dataset (n = 3611)	
	median or %	IQR*	median or %	IQR*

Male Gender	45.2%		45.6%	
Smoking status				
Current smoker	20.4%		20.7%	
Ex smoker	13.1%		13.8%	
Age (year)	57	21	57	21
Body mass index (kg/m^2^)	24.6	4.8	24.7	5.0
Years of diagnosed diabetes	5	10	5	9
Systolic BP (mmHg)	134	27	134	28
Diastolic BP (mmHg)	76	13	76	15
HbA_1c _(%)	7.3	2.2	7.4	2.2
Blood haemoglobin (g/L)	13.8	2.1	13.8	2.2
White blood cell count (10^9^/counts/L)	7.0	2.4	7.0	2.4
Spot urinary albumin creatinine ratio (mg/mmol)	2.0	10.1	2.0	10.0
eGFR (ml min^-1 ^1.73 m^-2^) ξ	104.9	43.0	104.9	43.5
LDL-C (mmol/l)	3.13	1.24	3.11	1.29
HDL-C (mmol/l)	1.24	0.45	1.25	0.45
Triglyceride (mmol/L)	1.37	1.08	1.39	1.12
Non-HDL cholesterol (mmol/L)	3.87	1.40	3.87	1.43
**Drug use at baseline**				
On diet treatment	10.2%		9.9%	
Use of oral anti-diabetic drugs	61.0%		60.6%	
Use of anti-hypertensive drugs	34.1%		33.3%	
Use of insulin	17.5%		17.4%	
Use of LLD§	11.6%		13.2%	
Use of ACEI or ARB¶	20.4%		20.1%	
**Complications at baseline**				
Retinopathy	25.4%		27.4%	
Sensory neuropathy	26.0%		25.7%	
Peripheral arterial disease	5.8%		5.9%	
History of stroke	3.6%		4.4%	
**Complications during follow-up**				
Coronary heart disease before heart failure during follow-up	4.4%		4.4%	
Heart failure hospitalisation during follow-up	3.8%		4.0%	
Death during follow-up	9.4%		9.9%	

*, Median (IQR = interquartile range); ξ, eGFR = estimated glomerular filtration rate; §, LLD, lipid lowering drugs; ¶, ACEI, Angiotensin-converting enzyme inhibitors and ARB for angiotensin II receptor blockers.

### Predicting models

In the training dataset, age, sex, BMI, HbA_1c_, Log_10 _(ACR+1) and blood Hb at baseline and CHD event during follow-up were selected by the model as predictors for HF-related hospitalisation. The functional forms of age, BMI, HbA_1c_, Log_10 _(ACR+1) and Hb seem adequate (p > 0.40). All the predictors apart from sex (p < 0.0001) did not violate the proportional hazards assumption (p > 0.20). To correct for violation of proportional hazards assumption in sex (p = 0.0080), sex-stratified Cox model was used to derive β and hazard ratio estimates of age, BMI, HbA_1c_, Log_10 _(ACR+1) and blood Hb at baseline and CHD during follow-up. Their hazard ratios (both univariate and multivariate analyses), β coefficients and 95% confidence intervals (CI) are listed in Table 2. Over-fitting of the final predicting model is, if any, very small (the shrinkage = 0.9744).

**Table 2:** Parameter estimates of the risk score for heart failure hospitalisation in the training dataset.

	Univariate Analysis		Multivariate Analysis			
Variables†	Hazard ratio (95% CI)	P value	Estimates of β values	S.E.M	Hazard ratio (95% CI)	P value

Variables used in the predicting model						
Age, per year	1.10(1.08–1.12)	<.0001	0.0709	0.0107	1.07(1.05–1.10)	<.0001
Body mass index, per kg/m^2^	1.01(0.97–1.05)	0.6568	0.0627	0.0286	1.07(1.01–1.13)	0.0282
Glycated haemoglobin, per %	1.16(1.07–1.25)	0.0003	0.1363	0.0550	1.15(1.03–1.28)	0.0132
Log_10_(1+spot urinary albumin to creatinine ratio, per mg/mmol)	4.50(3.65–5.54)	<.0001	0.9915	0.1409	2.70(2.32–3.55)	<.0001
Blood haemoglobin, per g/dL	0.61(0.55–0.68)	<.0001	-0.3606	0.0684	0.70(0.61–0.80)	0.0001
Coronary heart disease during follow-up (1. yes, 0. no)	3.61(2.24–5.81)	<.0001	0.8161	0.2675	2.26(1.26–4.05)	0.0061
Variables not used in the predicting model						
Duration of diabetes, per year	1.07(1.05–1.09)	<.0001			Not selected	
Glomerular filtration rate, per ml min^-1 ^1.73 m^-2^	0.97(0.96–0.97)	<.0001			Not selected	
Systolic blood pressure, per mmHg	1.03(1.02–1.04)	<.0001			Not selected	
Diastolic blood pressure, per mmHg	1.01(0.99–1.02)	0.5640			Not selected	
Low density lipoprotein cholesterol, per mmol/L	1.12(0.94–1.33)	0.2155			Not selected	
High density lipoprotein cholesterol, per mmol/L	0.80(0.48–1.34)	0.3976			Not selected	
Triglyceride, per mmol/L	1.04 (0.96–1.12)	0.3665			Not selected	
Smoking status					Not selected	
Current smokers	0.62(0.37–1.02)	0.0592				
Ex-smokers	1.96(1.28–3.01)	0.0020				
Never	1.0					
Use of oral anti-diabetic drugs at baseline	1.04(0.73–1.47)	0.8471			Not selected	
Use of anti-hypertensive drugs at baseline	3.98(2.79–5.67)	<.0001			Not selected	
Use of LLD at baseline	1.88(1.16–3.09)	0.0112			Not selected	
Use of ACEI or ARB at baseline	2.44(1.66–3.59)	<.0001			Not selected	
Use of insulin at baseline	3.59(2.52–5.10)	<.0001			Not selected	

†, Sex-stratified Cox model was used to obtain estimates of β values and hazard rato of predctors for heart failure. χ^2 ^test for likelihood ratio test = 234.60, Degree of freedom = 6, p < 0.0001 and the shrinkage of the model is = (234.60-6)/274.66 = 0.9744; Not selected indiates not selected by the backward algorithm at the significant levelt of 0.05.

CI, confidence interval; LLD, lipid lowering drugs; ACEI, Angiotensin-converting enzyme inhibitors; ARB, angiotensin II receptor blockers

Based on the values of β coefficients, the risk score and 5-year probability of HF that needs hospitalisation are constructed as follows:

HF-related hospitalization risk score = 0.0709 × age (year) + 0.0627 × BMI (kg/m^2^) + 0.1363 × HbA_1c _(%) + 0.9915 × Log_10_(1+ACR) (mg/mmol) - 0.3606 × Blood Hb (g/dL) + 0.8161 × CHD during follow-up (1 if yes).

The 5-year probability of heart failure = 1-S_0_(5)^EXP{0.9744 × (Risk Score - 2.3961)}^, where S_0_(5) = 0.9888 if male and 0.9809 if female.

### Validation of risk equations

**Figure 1:**
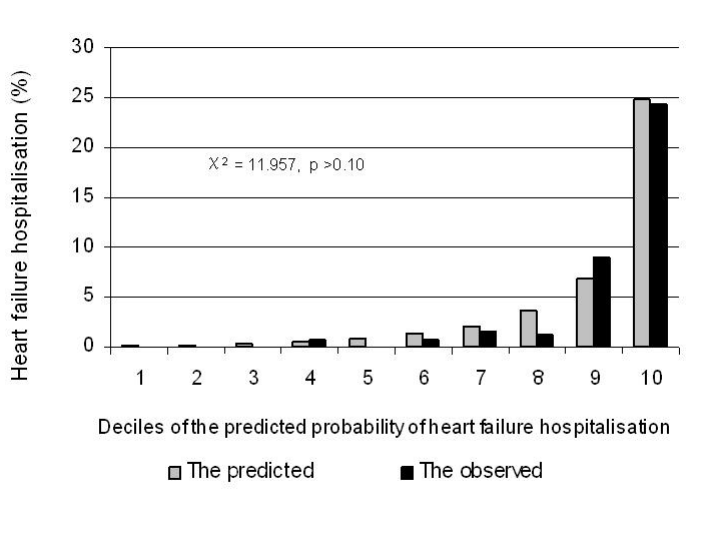
The predicted probability of heart failure that needed hospitalisation versus the observed probability over 5 years of follow-up in the test dataset.

In the test dataset, the predicted probability of HF-related hospitalisation over 5 years of follow-up was not significantly different from the observed probability of HF-related hospitalisation probability (p > 0.20) (Figure 1). The overall C index (for overall discrimination) was 0.853 (95% CI: 0.819–0.886). The follow-up time and censoring status adjusted aROC for 5 years of follow-up was 0.920. Using the cut-off point of ≥3.4683 for the risk score, the sensitivity was 90.5% and the specificity was 75.6%. If the cut-off point of 4.5334 was used, the sensitivity decreased to 71.1% while the specificity increased to 90.4%. Sensitivities and specificities of other cut-off points are listed in Table 3.

**Table 3:** Sensitivity, specificity, positive predictive values at selected risk scores and the probability of heart failure over 5 years of follow-up in the test dataset

Risk score cutoff point*	Predicted probability of heart failure		Sensitivity	Specificity	Positive predictive value	Negative predictive value
					
	Women	Men				
2.5606	0.0131	0.0129	0.980	0.571	0.429	0.086
3.0776	0.0216	0.0213	0.954	0.685	0.315	0.111
3.3153	0.0272	0.0267	0.929	0.726	0.274	0.122
3.4683†	0.0315	0.0310	0.905	0.756	0.244	0.132
3.5859	0.0353	0.0346	0.896	0.777	0.223	0.142
3.6385	0.0371	0.0364	0.883	0.786	0.214	0.145
3.9032	0.0477	0.0469	0.873	0.827	0.173	0.172
3.9644	0.0506	0.0497	0.851	0.837	0.163	0.177
4.1012	0.0576	0.0566	0.831	0.857	0.143	0.193
4.2309	0.0651	0.0640	0.821	0.877	0.123	0.216
4.3201	0.0708	0.0696	0.786	0.887	0.113	0.222
4.4208	0.0778	0.0765	0.727	0.894	0.106	0.220
4.5334†	0.0864	0.0849	0.711	0.904	0.096	0.234
4.6189	0.0936	0.0920	0.698	0.914	0.086	0.250
4.7414	0.1048	0.1030	0.662	0.923	0.077	0.261
4.8633	0.1172	0.1152	0.607	0.931	0.069	0.266
5.0962	0.1448	0.1424	0.551	0.950	0.050	0.311
5.4119	0.1916	0.1886	0.437	0.966	0.034	0.345

*, Calculated using the risk score equation; **, Calculated using the 5-year heart failure probability; †, The suggested cutoff points.

## Discussion

In this analysis, we demonstrated that HF that needed hospitalisation can be predicted with good accuracy in type 2 diabetes. Using 7 commonly collected variables: age, sex, BMI, HbA_1c_, ACR and blood Hb, the risk score can provide absolute risks of hospitalisation for HF, which depends on whether CHD event/s occurs or not in a 5-year period. The risk score has achieved an excellent discriminatory power of 0.920 for predicting hospitalisation due to HF over a 5-year period in type 2 diabetic patients while maintaining adequate calibration.

The discrimination of a risk score determines the efficacy and thus usefulness of a risk score. Several risk scores have been published for predicting hard CHD events, i.e. nonfatal myocardial infarction and fatal CHD events, in both general and type 2 diabetic populations. The Framingham CHD risk score has an C-statistics of 0.79 for men and 0.83 for women in their original population and 0.63–0.75 for men and 0.66–0.83 for women in other USA populations [20]. Liu *et al *[21]validated the Framingham risk score in a general Chinese population and the re-calibrated risk score had an aROC of 0.71 for Chinese men and 0.74 for Chinese women. The UKPDS risk engine for hard CHD endpoints did not report any measures of discrimination in the original report [4]. A validation study in a UK type 2 diabetic population by Guzder *et al *[22] reported that the UKPDS CHD risk engine had a discrimination of 0.67 for C-statistics. Donnan *et al *[23] developed a risk score for major CHD in type 2 diabetes population with a discrimination score of 0.71 in the original dataset and 0.69 in an independent dataset. Another risk score which predicted cardiovascular events and mortality using clinical predictors and baseline dobutamine stress echocardiography reported C-statistics values of 0.60 and 0.64 respectively [24]. Thus, a discriminatory power of C index 0.85 and aROC 0.92 of the current HF-related hospitalisation risk score indicates an excellent performance in distinguishing between those who would need hospitalisation for HF from their counterparts who would not. Comparing this risk score with other risk scores that were derived from the same cohort, the risk score has better discriminatory power than CHD (aROC: 0.74) [9], stroke (aROC: 0.77) [8] and all-cause mortality (aROC: 0.85) risk scores [10] but poorer discriminatory power than end stage renal disease risk score (aROC > 0.94)[6,7]. Similarly, the calibration is also adequate, better than the CHD risk score[9]but poorer than the all-cause mortality risk score [10].

Besides the CHD status on follow-up, the risk scores included as few as 6 baseline variables: age, BMI, HbA_1c_, ACR, blood Hb and sex. Most of these parameters have been verified to be associated with HF in other studies. HbA_1c _has been repeatedly found to be associated with increased risk of HF [1,25]. Low hematocrit and Hb levels have recently emerged as a major promoter of cardiovascular morbidity and mortality [26]. In agreement with Horwich *et al *[27] who reported association of anemia with early mortality in patients with advanced HF, we found that blood Hb had a strong independent predictive effect in the present cohort. Low Hb level can cause HF via many mechanisms including tissue hypoxia and left ventricular failure [28]. Several studies have now confirmed the strong risk association between albuminuria and HF in patients with type 2 diabetes [29-31]. Hockensmith *et al *[30] reported that baseline albuminuria was independently associated with 5.4 fold increased odds ratio of hospitalizations due to HF (95% CI: 2.3–12.5). In the Angiotensin II Antagonist Losartan Trial which recruited 1513 type 2 diabetic patients with nephropathy, high baseline albuminuria was associated with a 2.70-fold risk for HF compared with patients with low levels of albuminuria and that reduction of ACR at 6 months post treatment including the use of ARB predicted reduced risk of new onset of HF [32]. A study reported that BMI was not a significant predictor for mortality in patients with advanced HF [33]. In our analysis, BMI was also not a significant predictor of HF on univariable analysis but became significant after adjustment for Hb. Close associations between high blood Hb, hyperviscosity syndrome and obesity [34] may mask the true increased risk of high BMI if blood Hb was not adjusted.

The risk score used future CHD events as a "predictor". The future event is unknown at the time of prediction. Nevertheless, the CHD absolute risk can be estimated using the CHD risk score developed from the same cohort[9], using age, sex, current smoking status, duration of diabetes, non high density lipoprotein cholesterol, ACR and eGFR. In the UKPDS, both CHD and stroke risk engines were used in the case mortality risk engine to estimate the absolute risk of case mortality of patients who subsequently developed CHD or stroke event [35]. Similarly, our CHD risk score can also be used in the risk score to estimate the absolute risk of hospitalization for HF.

## Limitations

First, similar to another study [29], we used the definition of hospitalisation for HF rather than HF itself. The possibility underestimating severe HF incidence using this definition should be very small since Hong Kong Government maintains a heavily subsidised health care system and severe HF without being hospitalised are most unlikely. Second, although the registry is not population-based, due to the lack of a comprehensive health insurance policy and integrated primary health care system in Hong Kong, majority of patients in patients with chronic diseases such as diabetes and heart disease are managed in public hospitals. In 2000, Department of Health of Hong Kong conducted a survey and reported that over 90% of patients diagnosed with diabetes were managed in the public health sector [10]. Third, our cohort of type 2 diabetic patients had a wide range of disease duration which may theoretically widen the 95% CI of parameter estimations. On the other hand, it is the heterogeneity that extends usefulness of this risk score from predicting HF hospitalization at diagnosis to predicting HF hospitalisation during long-term care of patients with type 2 diabetes, i.e. the risk score can be used periodically during chronic care management.

## Conclusion and Implications

To our knowledge, this is the first risk score developed to predict HF events in a type 2 diabetic cohort. Given the rising prevalence of type 2 diabetes [36] and the high risk of morbidity and mortality of HF, especially in patients with diabetes [1], a risk score with such excellent performance, should enable clinicians to identify high risk subjects for intensive monitoring and treatment. Hence, validation of the risk score in other populations will be of public health importance.

## Abbreviations

ACEI: Angiotensin-converting enzyme inhibitor; ACR: Albumin: creatinine ratio; ARB: angiotensin II receptor blockers; aROC: Area under receiver operating characteristic curve; BMI: Body mass index; CHD: Coronary heart disease; eGFR: Estimated glomerular filtration rate; Hb: Haemoglobin; HbA_1c_: Glycated haemoglobin; HF: Heart failure; HR: Hazard ratio; UKPDS: United Kingdom Prospective Diabetes Study; SCR: Serum creatinine.

## Competing interests

The authors declare that they have no completing interests.

## Authors' contributions

XY performed the statistical analysis and drafted the manuscript. JC, RM, WS, GK, AK, CC and PT were involved in study design, coordination, data acquisition and manuscript revision. CL and CH facilitated retrieval of laboratory data and clinical outcomes. All authors have read and approved the final manuscript.
